# Natural T cell autoreactivity to melanoma antigens: clonally expanded melanoma-antigen specific CD8 + memory T cells can be detected in healthy humans

**DOI:** 10.1007/s00262-018-02292-7

**Published:** 2019-02-19

**Authors:** Anna Przybyla, Ting Zhang, Ruliang Li, Diana R. Roen, Andrzej Mackiewicz, Paul V. Lehmann

**Affiliations:** 1 Research and Development Department, Cellular Technology Limited (CTL), 20521 Chagrin Boulevard, Shaker Heights, Cleveland, OH 44122-5350 USA; 2 Department of Cancer Immunology, Chair of Medical Biotechnology, Poznan University of Medical Sciences, Poznan, Poland; 3 Department of Diagnostics and Cancer Immunology, Greater Poland Cancer Centre, Poznan, Poland

**Keywords:** CD8 + T cells, Melanoma antigens, ELISPOT, Stem-cell like CD8 + T cells, PIVAC 17

## Abstract

We used four-color ImmunoSpot® assays, in conjunction with peptide pools that cover the sequence of tyrosinase (Tyr), melanoma-associated antigen A3 (MAGE-A3), melanocyte antigen/melanoma antigen recognized by T cells 1 (Melan-A/MART-1), glycoprotein 100 (gp100), and New York esophageal squamous cell carcinoma-1 (NY-ESO-1) to characterize the melanoma antigen (MA)-specific CD8 + cell repertoire in PBMC of 40 healthy human donors (HD). Tyr triggered interferon gamma (IFN-γ)-secreting CD8 + T cells in 25% of HD within 24 h of antigen stimulation ex vivo. MAGE-A3, Melan-A/MART-1, and gp100 also induced recall responses in 10%, 7.5%, and 2.5% of HD, respectively. At this time point, these CD8 + T cells did not yet produce GzB (granzyme B). However, they engaged in GzB production after 72 h of antigen stimulation. By this 72-h time point, 57.5% of the HD responded to at least one, and typically several, of the MA. A closer characterization of the Tyr-specific CD8 + T cell repertoire indicated that it was low-affinity, and to primarily entail a stem cell-like subpopulation. Collectively, our data reveal pre-existing endogenous T cell immunity against melanoma antigens in healthy donors, and analogous to natural autoantibodies, we have termed this “natural T cell autoreactivity”.

**Electronic supplementary material:**

The online version of this article (10.1007/s00262-018-02292-7) contains supplementary material, which is available to authorized users.

## Introduction

Successful immune therapeutic approaches to melanoma [1] have drawn attention to the importance of monitoring melanoma antigen- (MA-) specific T cells. Melanoma is considered a highly immunogenic tumor [2, 3] with a T cell response to the tumor spontaneously developing as soon as it metastasizes into the draining lymph nodes [4]. It is thought that prior to this priming event, the MA-specific T cells are antigen-inexperienced/naïve [4] and like the naïve antigen-specific T cell repertoires in general, occur in frequencies far too low for detection. However, the opportunity to characterize MA-specific T cells increases after these T cells have been primed, clonally expanded, and differentiated [5]. For this reason, most successful efforts to characterize MA-specific T cells have focused on melanoma patients in whom the metastasizing tumor has already primed a T cell response. Consequently, there is relatively little information available on the MA-specific repertoire before this priming event, that is, in healthy subjects. This report aims at filling this gap. By characterizing the MA-specific T cell repertoire in healthy donors, we aim at establishing the baseline against which spontaneously-developing and experimentally induced immunity to melanoma can be compared.

The initial efforts to characterize MA-specific T cells not only focused on patients with metastasizing melanoma, but also involved protocols that aimed at expanding MA-specific T cells in vitro for several weeks before analysis. Frequently, these protocols involving repeated antigen stimulation cycles in the presence of growth factors [5, 6]. As the limit of detection using flow cytometry is roughly one antigen-specific T cell within 10,000 bystander cells (that is, 0.01%, [7]), this frequently used amplification step aims to enable detection of rare MA-specific T cells that would otherwise go undetected [5]. More importantly, CD8 + T cell subpopulations do not possess equivalent expansion rates [8, 9], and can alter their activation/differentiation status after antigen stimulation depending upon the culture conditions [10]. Therefore, concerned by altering T cell repertoires through in vitro culture, the field is moving to the analysis of T cells freshly isolated from blood, that is, ex vivo. In the present study, we performed ex vivo analysis of MA-specific T cells.

Enzyme-linked immunospot assay (ELISPOT) analysis has been gaining increasing popularity for ex vivo immune monitoring [11]. Unlike the multimer approach [12], ELISPOT assays are not limited to single peptides that are tailored to individual HLA alleles. Instead, extensive peptide libraries can be tested simultaneously as pools to systematically accommodate the array of potential antigenic determinants, irrespective of the test subject’s HLA type. Relatively little information is lost through pooling peptides since the numbers of cells recognizing the individual peptides contained in the pool add up to the number of cells activated by the pooled peptides [13].

For a long time, a weakness of ELISPOT assays *vs*. multimer analysis was that ELISPOT was suited only for single color/parameter analysis. Namely, counting the numbers of antigen-stimulated T cells in the test sample that secrete one analyte at a time, typically interferon-gamma (IFN-γ). In contrast, multimer-stained cells can be counterstained for expression of cell surface markers. Such multimer studies revealed that CD8 + T cells occur in subpopulations with fundamentally different surface phenotypes, translating into altered characteristics and contributions to host defense (summarized for cancer-relevant CD8 + T cell immunity in [8]). Ongoing efforts to characterize antigen-specific CD8 + T cells primarily focus on defining their surface phenotype, and in turn, seek to detail their activation/differentiation status. In the present study, we rely on a novel four-color ImmunoSpot® analysis approach to similarly define the effector capabilities of antigen-specific T cell subpopulations [14].

In the present study, we leverage the strength of ELISPOT to analyze the MA-specific T cell repertoire from healthy human donors (HD) directly ex vivo. First, we take advantage of the high sensitivity of ELISPOT. As performed, our assays are capable of enumerating individual MA antigen-specific CD8 + T cells within 250,000 PBMC [15]; corresponding to a detection limit of 0.003%. Second, we leverage the capacity of ELISPOT for assays using large peptide libraries. In this study, we utilize peptide libraries representing MA antigens, such as tyrosinase (Tyr), melanoma-associated antigen A3 (MAGE-A3), melanocyte antigen/melanoma antigen recognized by T cells 1 (Melan-A/MART-1), glycoprotein 100 (gp100), and New York esophageal squamous cell carcinoma-1 (NY-ESO-1). Each antigen is represented by an individual peptide library that systematically covers the entire amino acid sequence. All potential antigenic determinants of MA antigens are thus presented, irrespective of the HLA type of the donor and which HLA allele(s) serve as the restriction element(s). Third, we perform four-color ELISPOT assays to simultaneously measure IFN-γ, tumor necrosis factor alpha (TNF-α), IL-2, and GzB production. Collectively, the cytokine secretion profile of individual MA-specific CD8 + T cells defines their subpopulation lineage [14]. Finally, we measure the affinity [16] of MA-specific CD8 + T cells. By providing a comprehensive, high-resolution analysis of the MA-specific CD8 + T cell repertoire in HD, we introduce the feasibility of this approach and establish the baseline for immune monitoring of unvaccinated or vaccinated melanoma patients.

## Materials and methods

### PBMC donors

Peripheral blood mononuclear cells (PBMC) from healthy human donors (HD) were selected from the ePBMC library [Cellular Technology Limited (CTL), Shaker Heights, OH, USA]. The characteristics of the selected donors are specified in Suppl. Table 1. The PBMC were stored in the vapor phase of liquid nitrogen until testing. Processing (thawing, washing, and counting) of PBMC was done according to CTL’s protocols [17]. The viability of thawed cells exceeded 90% for all PBMC samples. The PBMC were resuspended at a final concentration of 2.5 × 10^6^ PBMC/ml in CTL-Test Medium (CTLT-005, from CTL) of which 100 µl (250,000 cells) was plated per well into ELISPOT assays.

### Antigens

Five melanoma-associated antigens were used for this study, all were purchased from JPT (Berlin, Germany): PepMix Human (tyrosinase)—PM-Tyr; PepMix Human (MAGEA3)—PM-MAGEA3; PepMix Human (Melan-A/Mart-1)—PM-MelA; PepMix (Melanocyte protein Pmel 17gp100)—PM-GP100; PepMix (NY-ESO-1)—PM-NYE. Peptide pools consisted of 15-mer peptides that systematically cover the entire amino acid (aa) sequence of the respective protein in steps of 11 aa. All peptide pools were tested at a final concentration of 1 µg/ml for each peptide, with all peptides within the pool being represented at equimolar ratios. CEFpp32 (Cytomegalovirus-, Epstein–Barr virus and Flu virus pool of 32 peptides) was used as a positive control for CD8 + T cell reactivity [18]. This peptide pool was derived from CTL (Cat #: CTL-CEF-002) and used at a final concentration of 0.25 µg/ml.

### Human single color IFN-γ and granzyme B ImmunoSpot® assays

Single-color enzymatic ELISPOT assays were performed to detect PBMC secreting IFN-γ (CTL, Cat #: HIFNG-1/5M) or granzyme B (CTL, huGzB) following the manufacturer’s recommendations. These two single color enzymatic assays were performed in an identical manner, except for the use of the respective capture and detection antibodies. Briefly, in the first step, the PVDF membrane was pre-coated with capture antibody overnight. Next, antigens were dissolved in CTL-Test Medium (CTL, Cat # CTLT-005) and plated in 100 µl per well. CTL-Test Medium and PBMC alone constituted the negative control wells, with CEFpp32 and CPI as positive controls [19]. The plates with the antigen were stored at 37 °C in a CO_2_ incubator until the cells were ready for plating. PBMC were added at 250,000 cells/well in 100 µl using wide-bore pipette tips. Plates were gently tapped on each side ensuring even distribution of the PBMC in the wells. The cells were incubated with the antigens for 24 h or 72 h, as specified, in a humidified incubator at 37 °C and 9% CO_2_. After the incubation time, cells were removed, detection antibody added, and enzymatic visualization of the plate-bound cytokine performed by enzyme-catalyzed substrate precipitation. The plates were air-dried prior to analysis. ELISPOT plates were analyzed using an ImmunoSpot® S6 Ultimate Reader from CTL. Spot Forming Units (SFU) were automatically counted by the ImmunoSpot® Software (CTL) using the Basic Count Suite.

### Human four-color ImmunoSpot® assay

To identify CD8 + T cell subpopulations by co-expression profiles of IFN-γ, GzB, IL-2 and TNF-α, four-color ImmunoSpot® assays [14] were performed (CTL, hT4001F) using PBMC with known IFN-γ reactivity from previous experiments. Cells were pre-stimulated for 72 h with antigen (tyrosinase or CEFpp32) in 24-well plates at 2 × 10^6^ cells/well in an incubator at 37 °C, 9% CO_2_. These antigens were used at final concentrations of 1 µg/ml and 0.25 µg/ml, respectively. Thereafter, the cells were transferred into a 96-well PVDF membrane plate with low auto-fluorescence (contained in the kit) that was pre-coated with the four capture antibodies. The cells were added at 3 × 10^5^ cells/well, along with Tyr or CEFpp32 antigens at the concentration specified above and anti-CD28 (0.1 µg/ml). After 24 h incubation at 37 °C, 9% CO_2,_ the plates were washed and then incubated for 2 h at room temperature with the respective detection antibodies: anti-IFN-γ (CTLhT02), anti-TNF-α (CTLhT13), anti-IL2 (CTLhT56), and anti-GzB (CTL-hT59). The plates were then washed and incubated with the tertiary solutions for 1 h at room temperature. Detection and tertiary solutions were filtered through a 0.22 µm filter prior to usage. Following completion of detection steps, plates were washed, dried, scanned, and analyzed using an ImmunoSpot® S6 Ultimate Analyzer.

### CD8 + and CD4 + T cell depletion

To establish whether the peptide-specific T cell response was present in the CD4 + or CD8 + lineage, CD4 + or CD8 + cells were depleted from PBMC using magnetic beads (Stemcell Technologies Inc, Canada). The depletion assay was performed according to manufacturer’s instructions. Unseparated PBMC, or CD4 + or CD8 + cell-depleted populations were then plated on 96-well ELISPOT plates at 2.5 × 10^5^ cells/well, and the assay performed as described previously.

### Statistical analysis

ELISPOT counts follow Normal distribution among replicate wells, which permits the utilization of parametric statistics for identifying positive responses [20]. Accordingly, the Student’s *t* test was used for comparing SFU in the three antigen-containing replicate wells vs. the spot counts in the medium control wells. A *p* value < 0.05 was considered as the cut-off for positivity, with an additional requirement that spot counts in antigen-stimulated wells exceed 10 SFU/well.

## Results

### Detection of melanoma antigen-specific T cells secreting IFN-γ in healthy human donors

PBMC of 40 healthy adult donors (HD) were randomly selected from CTL’s ePBMC library. The age, gender, and HLA-type of these donors are listed in Suppl. Table 1. Five melanoma antigens (MA) antigens were used for evaluating donor cell reactivity. Each of these antigens was represented by a pool of 15-mer overlapping peptides that cover the entire amino acid (aa) sequence of the respective proteins. Tyrosinase (Tyr), a protein of 529 aa was represented by 117 peptides. Melanocyte protein Pmel 17 gp100, is a protein of 661 aa length that was covered by 163 peptides. Melan-A/MART-1, a protein of 118 aa was covered by 27 peptides. Melanoma-associated antigen MAGE-A3, 314 aa long, was represented by 76 overlapping peptides. The NY-ESO**-**1 tumor antigen of the cancer/testis family, with a length of 180 aa, was covered by 43 peptides. Each peptide within the respective pool was present in equimolar ratio, and each peptide pool was tested at 1 µg/ml concentration with the PBMC present at 250,000 cells per well. Under these conditions, the number of peptide-triggered IFN-γ producing cells is enumerated as spot-forming units (SFU) within the 250,000 PBMC present per well, that is, the frequency of such antigen-specific T cells is equal to the SFU count/250,000.

In the first set of experiments, PBMC from 40 HD were exposed to the 5 MA peptide pools for 24 h or 72 h, during which IFN-γ production was measured in a standard ImmunoSpot® assay. In the absence of antigen stimulation, less than 5 cells per well spontaneously produced IFN-γ for each of the donors at either the 24 h or the 72 h time points. Thus, the medium background was less than 5 SFU/well. To meet the positivity criterion, the difference between the three replicate medium control wells tested and the three replicate wells containing the test MA peptide pool needed to reach a statistical significance of *p* < 0.05 in the Student’s *t* test. For increased stringency towards scoring as positive, we made an additional requirement that spot counts in antigen-stimulated wells exceed 10 SFU per well.

**Fig. 1:**
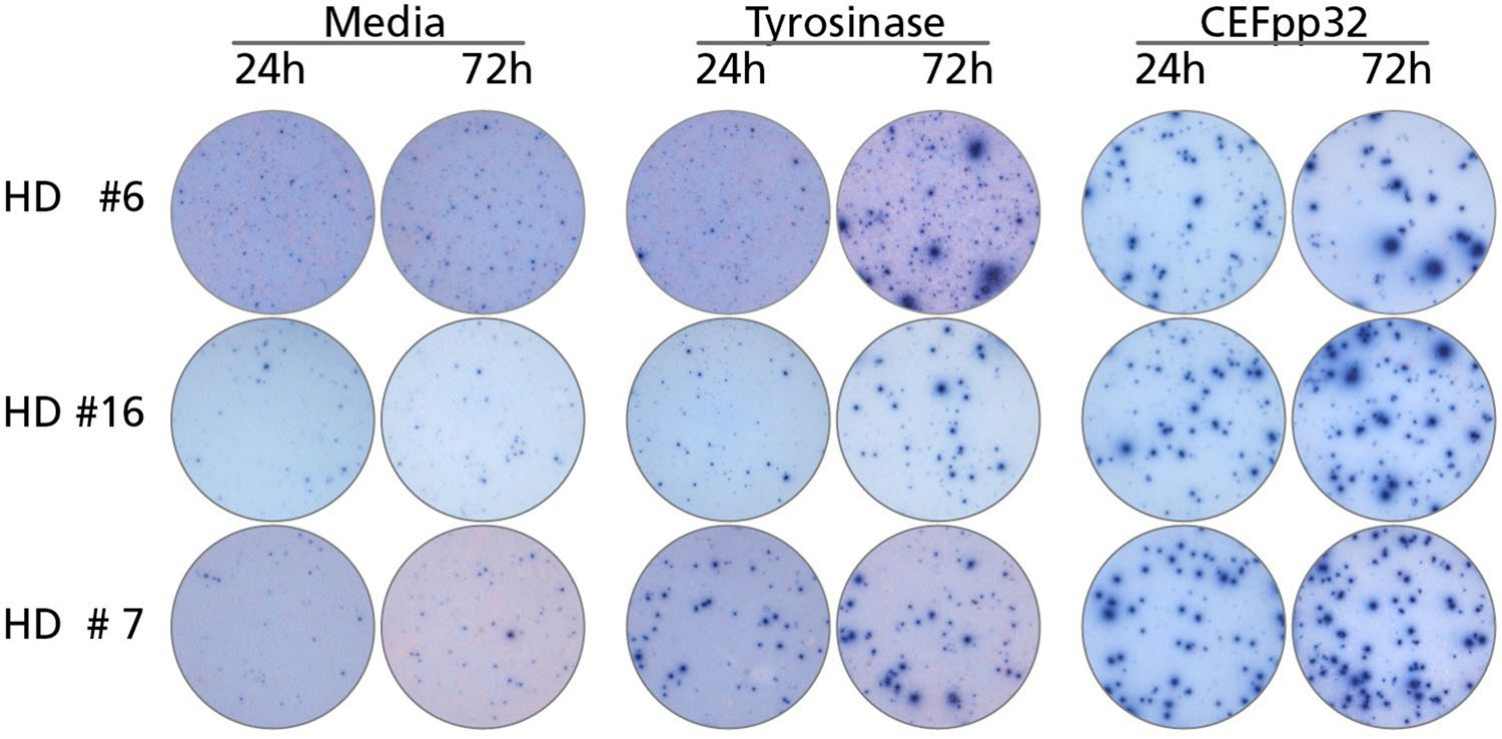
IFN-γ ELISPOTS induced by tyrosinase in PBMC of healthy donors (HD) within 24 and 72 h of antigen stimulation. Representative well images are shown for the data summarized in Table 1—the legend to Table 1 applies

Figure 1 shows representative examples of tyrosinase-induced IFN-γ spot formation after 24 and 72 h antigen stimulation. In some donors, for whom HD #7 is shown in this figure as an example, the tyrosinase peptide pool stimulated IFN-γ producing cells within 24 h. Nine of the 40 HD tested (22.5%) fell in this category (Table 1). Of note, in these donors the number of Tyrosinase-specific T cells capable of IFN-γ secretion was not substantially increased at the 72 h time point. Likewise, the number of CEF-triggered SFU did not differ much when measured at the 24 or 72 h time points (Fig. 1; Table 1). Moreover, the quality of such tyrosinase-induced spots (reflecting on the amount of IFN-γ secreted by the individual T cells) was similar to the spots triggered by the CEF peptide pool positive control. Strikingly, the numbers of spots induced by the tyrosinase peptide pool frequently reached the magnitude of the response triggered by the CEF pool. Thus, the frequency of tyrosinase-reactive T cells in the blood was frequently as high, or within an order of magnitude, as the CEF antigen-reactive repertoire. Therefore, tyrosinase-reactive T cells from these HD appear to be in vivo primed, clonally expanded, and cytokine-differentiated memory T cells.

**Table 1:** Melanoma antigen-triggered IFN-γ spot forming units in PBMC of healthy donors

Donor no *	Tyr	MAGE-A3	Melan-A/Mart-1	gp100	NY-ESO-1	CEFpp32
2	-/-	4/**32**	-/-	-/-	-/-	7/**11**
3	7/**22**	-/-	-/-	-/-	-/-	197/**255**
4	10/**17**	-/-	-/-	-/-	-/-	17/**22**
6	3/**22**	5/**16**	10/**13**	5/**16**	7/**13**	80/**79**
7	51/**72**	-/-	-/-	-/-	-/-	130/**170**
9	32/**42**	2/**13**	-/-	2/**13**	-/-	not tested*
10	-/-	1/**13**	-/-	-/-	-/-	not tested*
11	17/**20**	4/**14**	5/**20**	5/**13**	3/**11**	not tested*
13	-/-	3/**12**	-/-	-/-	-/-	64/**86**
14	-/-	5/**11**	-/-	-/-	-/-	9/**9**
15	-/-	1/**18**	-/-	-/-	-/-	15/**22**
16	12/**30**	76/**247**	2/**11**	-/-	-/-	88/**140**
17	38/**46**	-/-	-/-	-/-	-/-	86/**78**
19	-/-	3/**14**	-/-	-/-	-/-	38/**41**
20	-/-	15/**19**	-/-	-/-	-/-	5/**4**
21	27/**31**	-/-	-/-	-/-	-/-	301/**346**
23	-/-	-/-	2/**13**	-/-	-/-	3/**6**
24	7/**16**	31/**25**	10/**22**	8/**15**	4/**17**	125/**155**
26	16/**24**	-/-	-/-	-/-	-/-	215/**189**
27	-/-	3/**18**	6/**11**	-/-	-/-	5/**12**
31	3/**21**	27/**63**	11/**65**	24/**31**	-/-	40/**48**
32	14/**17**	-/-	-/-	-/-	-/-	87/**145**
36	39/**53**	-/-	-/-	1/**14**	-/-	122/**166**

PBMC (250,000 per well) of the 40 healthy donors specified in Suppl. Table 1 were exposed to the specified MA or the CEFpp32

Donors no*—only those donors who respond to at least one melanoma antigen

Not tested*—CPI was used as a positive control

A Student’s *t* test was done comparing SFU counts for the three antigen-induced replicates with the three replicates of the corresponding medium control. The table shows results for those donors only who scored at least one positive response. The first number represents the SFU counts at 24 h, the second number, in bold, at 72 h. SFU counts that did not reach statistical significance are represented by a hyphen

In other donors, for whom HD #6 and HD #16 are shown as examples in Fig. 1, the tyrosinase-induced production of IFN-γ was at or below the limit of detection. However, antigen-elicited IFN-γ production became clear cut by the 72-h time point. For tyrosinase, four of the 40 HD (10%) fell in this category (Table 1). Similar findings were made for the other MA as well: four of the 40 donors (10%) responded to MAGE-A3 within 24 h and the number of responsive donors increased to 14 (35%) by 72 h. In addition, the number of donors responding to Melan-A/Mart-1, gp100, and NY-ES0-1 increased from 7.5%, 2.5% and 0% at 24 h to 17.5%, 15% and 7.5% by 72 h, respectively (Table 1). Thus, after 72 h ex vivo, 57.5% of the HD tested exhibited a response to at least one, and typically several of the MA.

### Induction of granzyme B-secreting MA-specific T cells within 72 h ex vivo

**Fig. 2:**
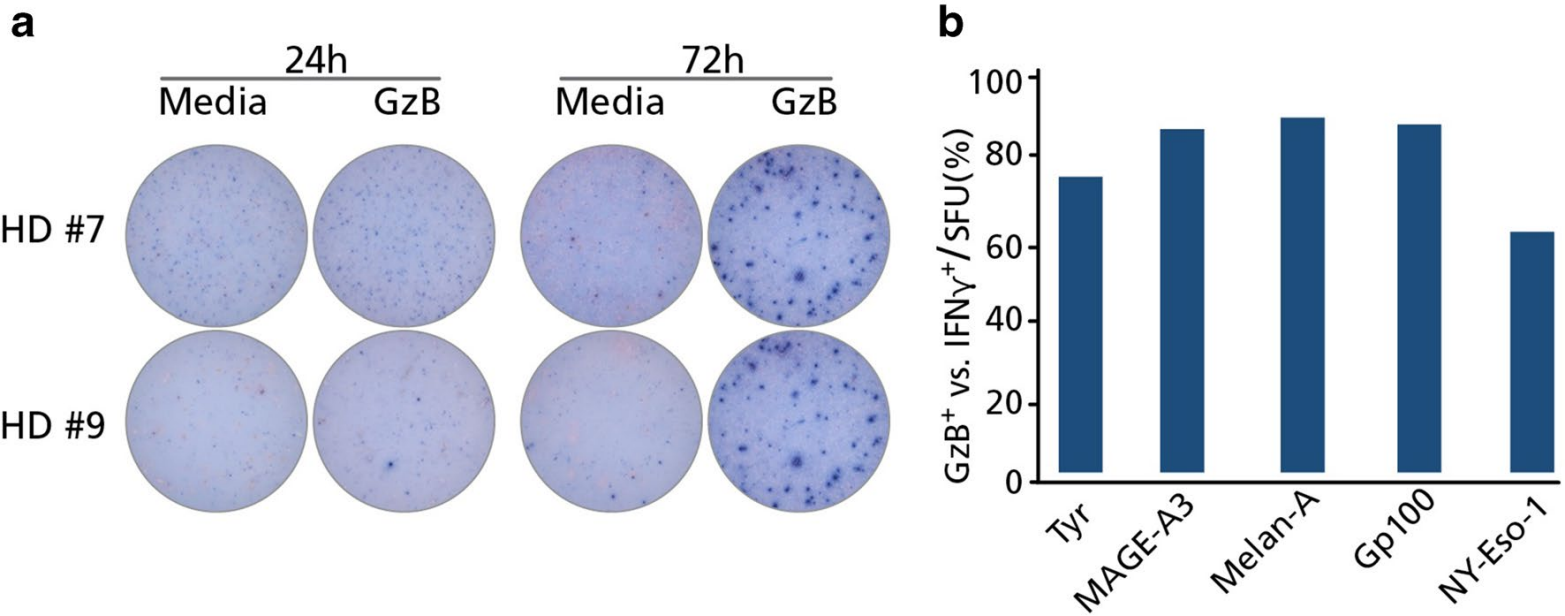
Granzyme B (GzB) production by PBMC of healthy donors after melanoma antigen exposure. The experiments were performed as detailed in the legend of Table 1 and in the text, except that GzB-secreting cells were detected. **a** Representative images are shown for two HD who responded with IFN-γ production to tyrosinase at the 24-h time point. **b** GzB positivity of IFN-γ positive cultures after 72 h of MA stimulation, after setting IFN-γ-positive MA-responses in each HD as 100% (see the 72 h IFN-γ data in Table 1). The percentage of these cultures that were also positive for GzB at the 72-h time point is depicted in the graph

Following 72 h of MA stimulation, a substantial IFN-γ response was observed using PBMC from multiple HD. Based on the number of spots, these responses most likely originate from clonally expanded, cytokine-differentiated memory CD8 + T cells. To verify this notion, we next tested whether MA-specific T cells could secrete granzyme B (GzB) at the 72-h time point; which is characteristic for effector CD8 + T cells capable of cytolysis [8, 21]. We, therefore, repeated the experiments above, exactly as preformed previously, but this time measuring GzB secretion by the PBMC. The PBMC of the same 40 HD were exposed to the five MA, again incubating the cells for 24 h or 72 h, and then the GzB response enumerated. At the 24-h time point, none of the MA peptide pools triggered GzB secretion in PBMC of HD. This also included the MA/HD combinations for which an IFN-γ response was detected at 24 h. Specifically, HD #4, #7, #9, #11, #16, #17, #21, #26, #32 and #36 (Table 1) all demonstrated IFN-γ production at 24 h in response to tyrosinase but were negative for GzB production. However, nearly all IFN-γ positive MA/HD combinations in Table 1 also exhibited strong GzB responses at the 72-h time point (Fig. 2a, b). Therefore, within 3 days of antigen stimulation ex vivo, MA-specific T cells convert into effector cells capable of secreting GzB. Moreover, MA-specific responses were observed irrespective of whether detectable IFN-γ secreting cells were already present at the 24-h time point.

### In HD the tyrosinase-induced IFN-γ SFU are produced by CD8 + T cells

**Fig. 3:**
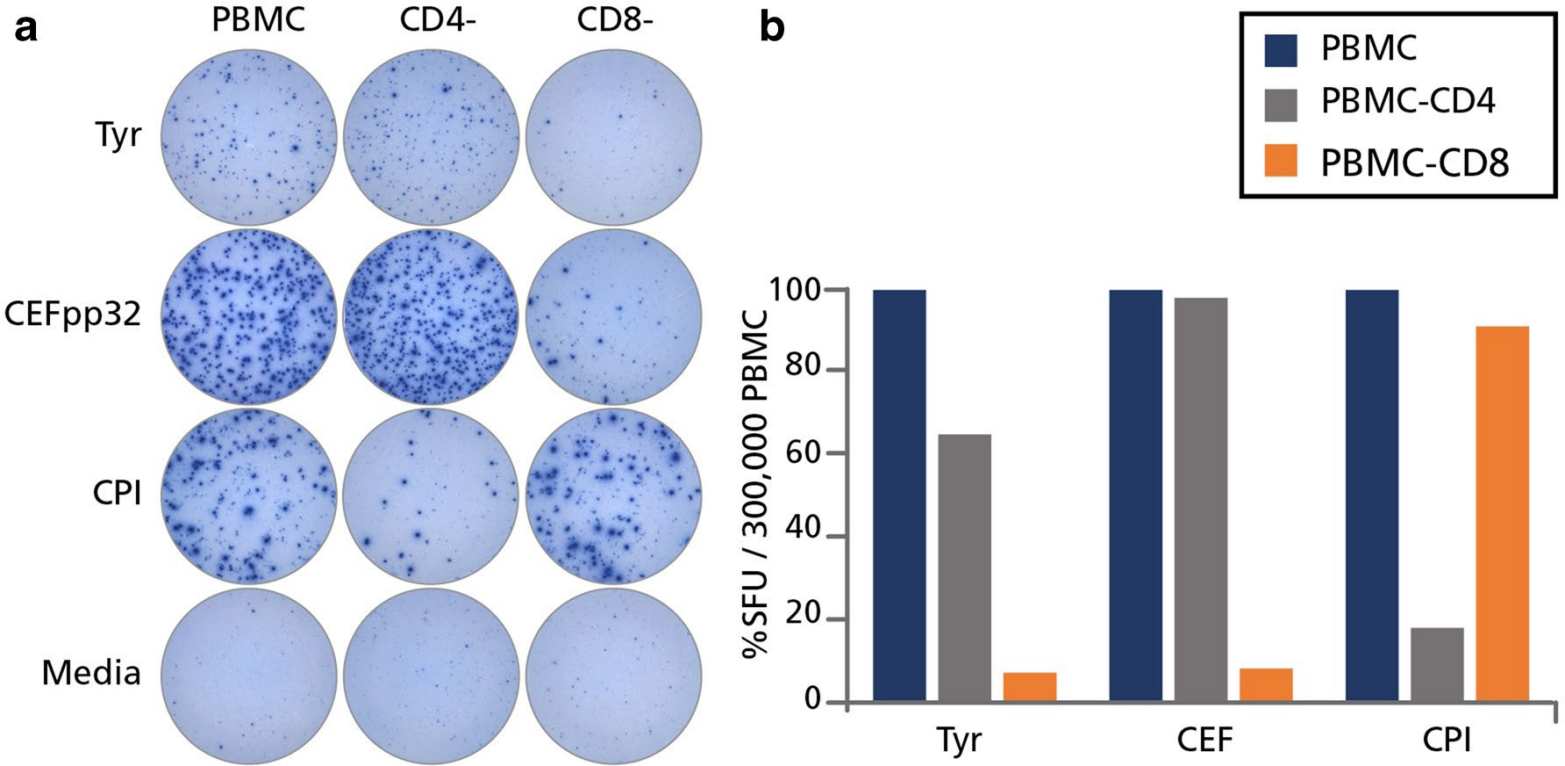
Tyrosinase-triggered IFN-γ spots are produced primarily by CD8 cells. PBMC from HD #36 were tested “unfractionated”, or after depletion of CD4 (CD4^−^) or CD8 (CD8^−^) cells via magnetic beads. These cells were then cultured either with tyrosinase (Tyr), CEF peptides (CEFpp32), CPI, or left unstimulated (media) and IFN-γ was detected after 24 h. **a** The original wells are shown. **b** The percentage reduction in spot forming unit (SFU) counts is shown after CD4 or CD8 cell depletion, representing the response of unfractionated PBMC as 100%

We next performed cell separation experiments to establish whether the MA-triggered IFN-γ spots originated from CD4 + and/or CD8 + T cells. Unseparated PBMC were tested along with PBMC that were subjected to CD4 + or CD8 + cell depletion. A representative experiment is shown in Fig. 3, in which CD8 + cell depletion nearly completely abrogated the response to the CEF peptide pool. Additionally, the magnitude of the CEF peptide response was largely unaffected by CD4 + cell depletion. Both of these observations are in line with the notion that the CEF responsive population are CD8 + T cells. In contrast, CD4 + cell depletion, but not CD8 + cell depletion, abrogated the recall response triggered by the CPI antigen pool encompassing proteins from cytomegalovirus, parainfluenza and influenza virus. Collectively, the CPI antigen and CEF peptide pools constitute positive controls for CD4 + and CD8 + T cell activation [19]. For tyrosinase, CD8 + cell depletion reduced SFU numbers in PBMC over 90% in some instances, whereas CD4 + cell depletion resulted in a smaller, 40% reduction. Therefore, the tyrosinase reactive IFN-γ secreting cells recalled by the 15-mer peptide pool were predominantly CD8 + T cells; however, there was also a significant percentage of CD4 + T cells that were responsive to this antigen. While the CD4+/CD8 + cell lineage of the T cells responding to the other 4 MA were not formally evaluated, the induction of GzB after 72 h (see Fig. 2b) suggests that these responses also entail a major CD8 + T cell component.

### Characterization of the tyrosinase-reactive CD8 + T cell lineages in HD

**Fig. 4:**
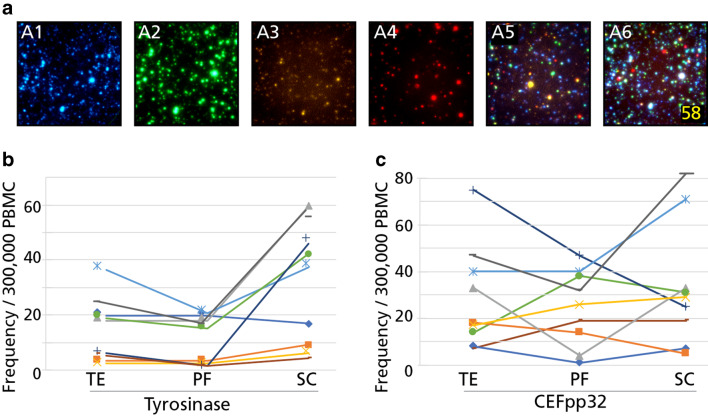
Lineage characterization of tyrosinase- and CEF peptide-reactive CD8 cells of healthy donors. **a** Representative wells of the individual color planes are shown in A1-A4, for IL-2, IFN-γ, TNF-α, and GzB, respectively. A5 depicts the overlay of the four individual color planes, and polyfunctional cells positive for all four analytes are highlighted in A6. Terminal effector cells (TE) were defined as IL-2^−^, IFN-γ^+^, TNF-α^+^, and GzB^+^; polyfunctional cells (PF) as IL-2^+^, IFN-γ^+^, TNF-α^+^, and GzB^+^, and stem cell-like cells (SC) as IL-2^+^, IFN-γ^−^, TNF-α^−^, and GzB^−^. PBMC of tyrosinase-reactive HD (donor #2, #7, #9, #16, #17, #21, #31 and #36) were cultured with the tyrosinase (**b**) or the CEF peptide pool (**c**) for 72 h, after which they were plated into a four-color ImmunoSpot® assay for 24 h, enabling detection of cells secreting IL-2, IFN-γ, TNF-α, and GzB

Cytokine co-expression patterns permit segregation of CD8 + T cells into sub-lineages [8]. Terminal effector cells (TE) co-express IFN-γ, TNF-α and GzB, but do not secrete IL-2. Polyfunctional CD8 + T cells (PF) secrete all four of these cytokine analytes, while stem-cell like (SC) T cells secrete IL-2 in isolation. To determine which of the above CD8 + T cell sub-lineages encompassed the tyrosinase-specific response observed in HD, we performed four-color ImmunoSpot® assays studying the co-secretion of IFN-γ, TNF-α, GzB, and IL-2 by individual antigen-stimulated cells [14]. In parallel, CEF peptide pool-reactive CD8 + T cells were tested for comparison and the results are shown in Fig. 4. In five of nine HD tested, the tyrosinase-specific CD8 + T cells were predominantly stem cell-like T cells (SC: IL-2^+^/IFN-γ^−^, TNF-α^−^ and GzB^−^), and outnumbered the polyfunctional (PF: IL-2^+^/ IFN-γ^+^, TNF-α^+^ and GzB^+^) and terminal effector cells (TE: IL-2^−^/ IFN-γ^+^, TNF-α^+^ and GzB^+^) by at least twofold. The parallel characterization of CEF-reactive CD8 + T cells from these same nine HD showed no bias towards the SC phenotype, and instead PF and TE subpopulations were comparably represented.

### Characterization of the affinity of tyrosinase-specific CD8 + T cells in HD

**Fig. 5:**
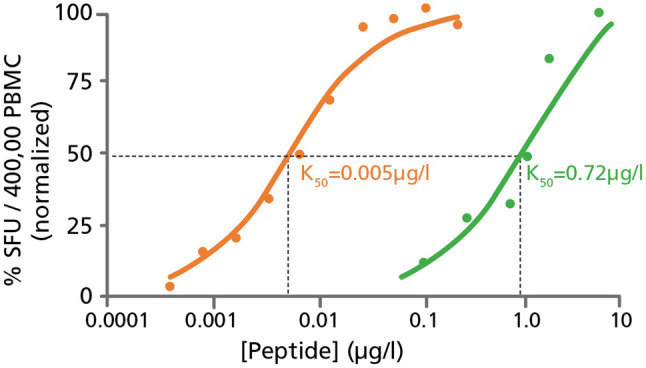
Tyrosinase-specific T cells in healthy donors are of low-affinity compared to CEF-specific T cells. Tyrosinase (green) and CEF (orange) peptides were serially diluted and used for testing at the specified concentrations using PBMC from HD #17; who was shown to respond to both peptide pools (refer to Table 1). Taking the SFU counts elicited at 10 µg/ml of peptide as 100%, the % response induced by the specified peptide concentrations is shown, and the peptide concentration that elicited 50% maximal stimulation was established as the *K*_50_ value

By titrating the antigenic peptides, one can establish the functional affinity, that is, the threshold for antigen-specific T cell receptor stimulation. High-affinity T cells respond to lower concentrations of peptide, whereas low-affinity T cells require higher concentrations of peptide to become activated [16]. Using this approach, we showed, for example, that negative selection in the thymus trims the high-affinity end of the autoantigen-specific T cell repertoire while sparing T cells with low affinity for the autoantigen [16]. To establish the functional affinity of the tyrosinase-specific CD8 + T cells in HD, donors displaying an IFN-γ response within 24 h were re-tested in an IFN-γ assay using titrated quantities of the tyrosinase peptide pool. Response curves to the tyrosinase and CEF peptide pools are depicted in Fig. 5 for a representative HD. The 50% maximally stimulatory tyrosinase peptide concentration, representing the *K*_50_ value, was 0.72 µg/ml for this particular HD. By contrast, CD8 + T cells specific for the CEF peptides were stimulated using a 200-fold lower peptide concentration (*K*_50_ = 0.005 µg/ml). These peptide titrations studies indicate that tyrosinase-specific CD8 + T cells exhibit low functional affinity for tyrosinase peptides, while CEF-specific CD8 + T cells display a high functional affinity for their cognate peptide.

## Discussion

The present study was designed to address current technical challenges for ex vivo assessment of T cells in general, and MA-specific T cells in particular. This initial study serves as a baseline assessment using healthy donors, and building upon these data, eventually has application to characterizing T cell reactivity in melanoma patients. In contrast to previous studies that predominantly utilized select MA peptides in the context of specific HLA alleles and the flow cytometry-based multimer approach, we opted to utilize extensive peptide pools that systematically covered the entire amino acid sequence of the melanoma antigens; tyrosinase, MAGE-A3, Melan-A/Mart-1, gp100 and NY-ESO-1. The first surprising finding was that 23 of 40 healthy donors (58%) displayed ex vivo reactivity to at least one of these MA antigens (Table 1). Eight of these HD responded to two or more MA. Specifically, two HD responded to two MA pools and another two HD responded to three MA pools. One HD responded to four MA, and the remaining three responded to all five MA peptide pools, respectively. NY-ESO-1 and gp100, while frequently recognized in combination with other MA, were not targeted in isolation. Overall, the MA-specific repertoire in HD appears to be diverse, and capable of recognizing each of five MA used in this study. Collectively, this observation justifies the use of all five of these peptide pools for comprehensive immune monitoring, and indicates that additional MA should be considered for assessment of T cell reactivity.

The present study also illustrates the feasibility of such a broad coverage of multiple antigens. As performed, using 250,000 PBMC per well and three replicates for each of the five MA plus the medium control, only 5.4 million PBMC were required to establish the breadth of the MA-specific repertoire from individual HD. Importantly, while this broad screening was performed using a single color IFN-γ assay, the same number of PBMC could have been used to define the respective MA-specific T cell subpopulations using a 4-color four-color ImmunoSpot® assay. Thus, even after accounting for the limiting PBMC numbers that can be obtained from melanoma patients (e.g. 20 million PBMC), the number of MA peptide pools that could be readily tested can be increased to 25 when tested in triplicate, or 80 when tested as single replicates; which might well be permissible as hardly anyone runs samples in flow cytometry in replicates.

Using the ELISPOT platform for measurement of T cell responses against “foreign antigens”, such as viruses, one can unambiguously identify donor cohorts that have, or have not, been exposed to a particular antigen. Such studies demonstrate that specific viral peptides, or peptide pools, elicit T cell-derived IFN-γ production in excess of the medium background using PBMC from virus-exposed cohorts but not in virus-naïve individuals [22]. Confirming this notion, when we recently tested similar peptide pools that cover HCMV antigens, we found that IFN-γ SFU were elicited only in CMV-exposed donors, but not in HCMV-negative individuals [23]. A similar experiment is detailed in Suppl. Fig. 1, which further illustrates this important point. In this study [23], and in the data presented in Suppl. Fig. 1, HCMV peptide pools that represent 11 distinct proteins encoded by of HCMV virus, each consisting of 15-mer peptides that span the entire length of the respective aa sequences in steps of 11 aa, were used for stimulation. These HCMV peptides were from the same manufacturer, JPT, and were synthesized and handled under identical experimental condition, including being used at an equivalent concentration as the MA peptides reported here. Each of the three HCMV-infected donors shown in Suppl. Fig. 1 (donors A, B and C) responded vigorously to most of the HCMV antigens/peptide pools tested. In contrast, none of the three HCMV negative HD (donors D, E, and F) responded to any of the 11 HCMV peptide pools with significant SFU numbers over the medium background. Thus, in a situation in which HCMV infection, and hence the development of an immune response, can be confirmed, the exquisite specificity of such peptide pools for detecting ex vivo-expanded memory T cells can easily be verified. However, since MA are ubiquitously expressed self-antigens, they may be more likely to induce immunologic tolerance than trigger clonal expansions and cytokine differentiation in healthy donors. Nevertheless, MA may be unique self-antigens since environmental factors such as sunburn, which promotes a pro-inflammatory environment, may enhance their immunogenicity and trigger initiation of an immune response.

The relatively high frequency of MA-specific CD8 + T cells detected amongst PBMC from HD was not anticipated. For example, under the 24-h recall conditions (Table 1), tyrosinase-reactive T cells in HD #7, #9, #17, and #36 reached or exceeded 30 SFU per 250,000 PBMC, which is a precursory frequency of 1:8300 or 0.008%. In three additional HD (#6, #24 and #31), MAGE-3A reactive cells were also present in this frequency range. While still at the detection limit of flow cytometry, the magnitude of these recall responses in an ELISPOT assay are typical of clonally expanded memory/effector T cell populations [24]. To this end, the frequency of MA-reactive CD8 + cells detected in these HD were in the range of clonally expanded memory cells, and were substantially greater than the < 1 in 250,000 PBMC frequency anticipated in the naïve T cell compartment.

Using HLA-A2*01 tetramers, Melan-A _(26−36)_ peptide-specific CD8 + T cells were detected at an atypically high frequency (0.07%) in HD, and these T cells were apparently naïve since they exhibited a CD45RA^+^ surface phenotype [25]. As such a high frequency of naïve T cells is unique, it is thought that these cells emerge through a particularly productive thymic selection process. As noted above, we have detected tyrosinase and Melan-A reactive CD8 + T cells at frequencies around 0.01% following 24 h antigen stimulation directly ex vivo. After 72 h of antigen stimulation, 8 of 40 HD responded to at least one of the MA at this relatively high, > 30 SFU per 250,000 PBMC or ~ 0.008%, frequency range. Altogether, 58% of HD displayed a significant response to at least one of the five MA antigens used for testing.

In general, peptide-specific naïve T cells occur at very low frequencies; < 1 per 250,000 PBMC. Additionally, naïve T cells do not secrete IFN-γ within the first 24 h of antigen stimulation [10]. While naïve T cells are capable of extensive proliferation following antigen recognition, the 24-h incubation period of an ELISPOT assay is sufficient for only a single cell division cycle. Thus, detection of MA-reactive T cells capable of IFN-γ production within a 24-h assay (Table 1; Fig. 1) signifies that these T cells have most likely undergone previous expansion and differentiation into Th1-type memory cells in vivo. Since the MA-specific T cells do not secrete GzB within the first 24 h of stimulation (Fig. 2b), these cells most likely represent resting (central) memory cells directly ex vivo. Following continued antigen stimulation, these cells go on to acquire expression of cytolytic granules and convert into GzB + effector CD8 + T cells capable of both cytolysis and IFN-γ secretion. The observed behavioral pattern of these MA-reactive T cells is typical of resting CD8 + T cells specific for virus antigens, including CEF peptide-reactive CD8 + T cells [21].

In this study we also identified MA-specific T cells from several HD that failed to secrete IFN-γ, along with GzB, after 24 h of antigen stimulation. However, these same donor cells yielded both IFN-γ and GzB producing cells after 72 h of stimulation with MA (Table 1; Fig. 1). Such cells likely represent progeny of stem cell-like (SC) CD8 + memory T cells [9], which can engage in rapid proliferation and then differentiate into effector cells capable of both IFN-γ and GzB expression following a longer 72 h antigen stimulation. Another possible explanation for the delayed cytokine secretion phenotype is that MA-specific T cells are naive at isolation. While self-antigen is likely to deplete the high-affinity end of the autoreactive T cell repertoire [16], T cells are positively selected on self-antigens and ongoing T cell receptor engagement is required for survival [26]. Therefore, the increased frequency of naïve MA-specific T cells could be attributed to an increased abundance of low-affinity, autoreactive T cells relative to those recognizing foreign antigens with high-affinity.

Data consistent with the notion that self-reactive T cells in HD can possess a unique phenotype has also been reported for cytokeratin-18-specific CD8 + T cells [27]. Such cells have undergone extensive clonal expansion in vivo, but are functionally unresponsive and do not secrete cytokine in response to antigen encounter directly ex vivo. However, after antigen stimulation these cells regain their functionality. The MA-reactive T cells from HD that failed to secrete cytokines at 24 h, but which became positive for cytokine secretion at 72 h, therefore, might be the progeny of such clonally expanded autoreactive T cells. Overall, these findings suggest that in the context of comprehensive immune monitoring it is not advisable to restrict assessment of T cell function(s) to a single set time point following antigen stimulation. Instead, it would be preferable to evaluate T cell recall responses over extended time frames [28]. Importantly, this approach is technically feasible using the ELISPOT platform in spite of limiting PBMC acquired from patients. Furthermore, since ELISPOT is a non-destructive technique and T cells survive the assay largely unharmed, PBMC can theoretically be transferred from one assay into the next, facilitating testing of the same PBMC [29].

Initial efforts reported in the literature that sought to identify melanoma-specific T cells required over a week of in vitro expansion [5]. To avoid this in vitro expansion step, and to enable determination of MA-specific T cell precursor frequencies as they exist in vivo, we performed standard 24-h recall assays. This approach is commonly utilized to identify in vivo primed memory T cells. In an ELISPOT assay that does not require signal enhancers, the 24-h antigen-stimulation period is optimal for detecting peptide-elicited cytokine production by CD8 + memory T cells [28]. Since resting memory CD8 + T cells do not possess cytolytic granules immediately following antigen stimulation, but instead acquire this effector function within 3–4 days after antigen stimulation [21], we extended our in vitro culture to 72 h to determine whether the resting memory cells detected at 24 h could differentiate into cytolytic effectors. To enable segregation of such effector T cells into distinct sub-lineages, we also performed 4-color ImmunoSpot® analysis at the 72-h time point. The closer characterization of cytokine expression profiles by tyrosinase-specific CD8 + T cells demonstrate that, in addition to terminal effector cells (TE: IL-2^−^, IFN-γ^+^, TNF-α^+^ and GzB^+^), the polyfunctional (PF: IL-2^+^, IFN-γ^+^, TNF-α^+^ and GzB^+^) and stem-cell like (SC: IL-2^+^, IFN-γ^−^, TNF-α^−^ and GzB^−^) lineages were also present within the MA-specific CD8 + T cell repertoires, with the latter stem cell-like subset prevailing (Fig. 4). The abundance of stem cell-like CD8 + T cells we observed serves as indirect evidence that tyrosinase-specific CD8 + T cells in HD have not engaged in prolonged immune encounters in vivo. In contrast, in cancer patients, due to T cell exhaustion resulting from persistent antigen stimulation, senescent and dysfunctional tumor antigen-specific CD8 + T cells seem to prevail, with a concomitant reduction in the stem cell-like memory cell pool [8, 30].

Titration of the antigenic peptides permits determination of the functional affinity of antigen-specific T cells [16]. We found the tyrosinase-specific CD8 + T cells in HD to be of relatively low-affinity (*K*_50_ ~ 1 µg/ml) compared to CEF-specific CD8 + T cells (*K*_50_ ~ 0.005 µg/ml). Unlike viral antigen-specific CD8 + T cells which recognized foreign peptide determinants, T cells recognizing self-antigens must somehow evade negative selection. To this end, our data suggest that the tyrosinase-specific CD8 + T cells we detected in HD escaped negative selection due to their low affinity for this self-antigen. In contrast, T cells bearing receptors that recognize tyrosinase peptides with high-affinity are likely to be purged from the repertoire through clonal deletion.

The data presented in this study also highlights the feasibility of evaluating the MA-specific T cell repertoire using a novel modification of the ImmunoSpot® technology. The sensitivity of the ELISPOT assay enables detection of low abundance T cells, far below the < 0.01% frequency range, which is not reliably achieved using a flow cytometry approach. The use of MA peptide pools also permitted parallel assessment of all potential antigenic determinants of the MA, which is not technically feasible using multimers. Studies of the activation kinetics also revealed an apparent naïve/anergic state of MA-specific T cells in vivo. Unlike flow cytometric assessment of cytokine production, which requires fixation and membrane permeabilization of the sample, cell viability is maintained during the ELISPOT and secondary assays can be performed without requiring additional cell material. Moreover, multi-color measurements of cytokine expression profiles using the 4-color ImmunoSpot® assay also permitted identification of the respective memory lineages and fractionation into stem cell-like, polyfunctional or terminal effectors cells. Finally, measurement of functional affinity was also deduced through peptide titration experiments, and was accomplished using fewer cells and requiring considerably less labor compared to flow cytometry. Importantly, all of the assays detailed in this report were accomplished using less than 20 ml of blood per donor, and with relatively minor investment of investigator effort and cost. Furthermore, all of the reported ELISPOT assay variants can be readily validated and adapted for generating data in a regulated environment [11]. Thus, we believe, these novel extensions of the ImmunoSpot® technology will largely facilitate efforts to better understand tumor antigen-specific T cells in health and disease, including their mobilization for treatment of tumors.

Melanoma become immunogenic in the metastatic stage, and the T cell response to the malignant cells has been quite well-characterized. The T cells in melanoma patients target non-synonymous mutations on various proteins and/or recognize overexpressed non-mutated melanoma antigens [2, 31, 32]. However, it was not apparent prior to our findings that benign melanocytes were capable of triggering endogenous T cell responses against melanocyte autoantigens which are neither overexpressed, nor mutated, and as such qualify as “self-antigens”. Our data draw attention to the existence of these melanocyte-specific CD8 + T memory cells in healthy human donors. Likely, this natural T cell autoreactivity gets primed during sunburns, when melanocyte activation occurs in the context of skin inflammation. At present, one can only speculate about the immunobiological significance of natural CD8 + T cell autoreactivity. One possible interpretation of this finding is that natural T cell autoreactivity to melanocyte antigens helps protects against development of melanoma. This notion is supported by ongoing studies, in which we found that 23 of 24 melanoma patients tested lacked MA reactivity that was demonstrated in this report using HD, while still exhibiting robust T cell reactivity against additional recall antigens (Anna Przybyla and Paul V. Lehmann—manuscript in preparation). A similar observation has been reported for breast cancer. Healthy women displayed high levels of spontaneous T cell autoreactivity to HER-2, while women with breast cancer were found to selectively lack such T cell responses [33]. One possible interpretation of such findings is that individuals lacking natural T cell autoreactivity to a given tumor are at increased risk of developing that cancer. However, there is an equally likely interpretation for the selective absence of natural T cell autoreactivity in cancer patients. Specifically, these pre-primed T cell populations may become “burned out” and undergo senescence in the face of ongoing antigen stimulation [34]. Such senescent T cells, while undetectable in an IFN-γ recall assay, could even promote the growth of the tumor [8, 30]. In either case, natural T cell autoreactivity targets autoantigens, and expression levels thereof, that are present on melanocytes. Once melanoma cells arise in the body, they offer a new set of target antigens for CD8 + T cell recognition, which can either be mutated or overexpressed melanoma antigens. The latter, though qualitatively unchanged relative to “self”, become targeted because they are presented at much higher MHC-peptide density on tumor cells. When expressed at a sufficiently high copy number on transformed melanocytes, low-affinity autoreactive T cells will likely receive sufficient TCR stimulation that their activation threshold is exceeded. By contrast, these same low-affinity autoreactive T cells would otherwise be ignorant of the same MHC-peptide combination when expressed at physiologic levels on normal melanocytes [35]. It is likely that checkpoint inhibitors may act on tumor-specific T cells that recognize mutated self-peptides, but it remains unclear whether such biologics also enhances the activity of pre-existing, natural T cell autoreactivity. Furthermore, the role of natural CD8 + T cell reactivity and prevention of melanoma in HD is presently unknown. Whether these cells are inactive in melanoma patients, or if they contribute to promoting the immunosuppressive tumor micro-environment after undergoing senescence are also key questions that will require further study.

### Electronic supplementary material

Below is the link to the electronic supplementary material.

Supplementary material 1 (PDF 573 KB)

## Abbreviations

CEFpp32: Cytomegalovirus-, Epstein–Barr virus and Flu virus pool of 32 peptides
CTL: Cellular Technology Limited
gp100: Glycoprotein 100
GzB: Granzyme B
HD: Healthy donor
IFN-γ: Interferon gamma
IL-2: Interleukin 2
MA: Melanoma antigen
MAGE-A3: Melanoma-associated antigen A3
Melan-A/Mart-1: Melanocyte antigen/melanoma antigen recognized by T cells 1
NY-ESO-1: New York esophageal squamous cell carcinoma-1
SC: Stem-cell like T cells
SFU: Spot forming unit
TNF-α: Tumor necrosis factor alpha
Tyr: Tyrosinase
