# Supplementary material for: Natural T cell autoreactivity to melanoma antigens: clonally expanded melanoma-antigen specific CD8 + memory T cells can be detected in healthy humans
Source: Cancer Immunol Immunother. 2019 Feb 19;68(5):709–20. doi: 10.1007/s00262-018-02292-7 (PMC11028361; doi:10.1007/s00262-018-02292-7)
Supplement: Supplementary file 1 — Supplementary material 1 (PDF 573 KB) [file 262_2018_2292_MOESM1_ESM.pdf]

Supplementary data

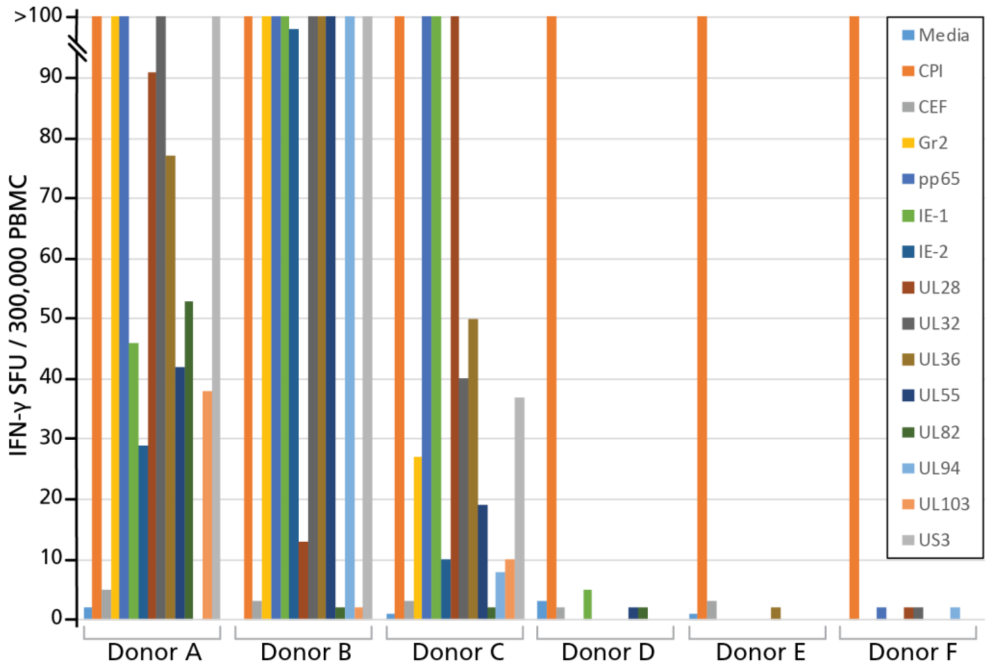

Supplementary Figure 1. HCMV peptides elicit IFN-γ SFU only in HCMV-infected donors A, B and C, but not in HCMV-negative donors D, E and F. Three HD who were seropositive for HCMV (Donor IDs A, B and C) and three HD who were seronegative for HCMV (Donor IDs D, E and F) were selected from the ePBMC database, and tested for IFN-γ SFU formation induced by HCMV grade 2 antigen (Gr 2, representing UV-inactivated HCMV virus, the yellow bars) as well as 11 peptide pools that cover 11 HCMV proteins (pp65, IE-1, IE-2, UL28, UL32, UL36, UL55, UL82, UL94, UL103, and US3, specified by color in the insert). CPI (the orange bars) and the CEF peptide pool (the grey bars) were tested as the positive control for stimulation of CD4 and CD8 cells, respectively. The media control is shown by the blue bar, first on the left of each panel. The SFU counts are capped off at 100 per 300,000 cells tested so that the lack od SFU induction by all HCMV antigens can be clearly seen for the HCMV negative donors D, E and F, contrasting the high counts induced in all three HCMV positive donors A, B and C.

Supplementary Table 1. Characteristics of healthy donors (HD) tested in this study.

			HLA Class 1		
Donor #	Age	Gender	HLA-A	HLA-B	HLA-C
1	35	Male	A*29:02<br>A*68:01	B*40:08<br>B*44:03	C*03:04<br>C*16:01
2	38	Male	A*30:02<br>A*33:03	B*18:01<br>B*53:01	C*04:01<br>C*07:01
3	24	Male	A*24:02<br>A*31:01	B*40:02<br>B*51:01	C*03:04<br>C*15:09
4	49	Female	A*01:01<br>A*01:01	B*14:02<br>B*41:02	C*07:01<br>C*17:03
5	27	Female	A*01:01<br>A*68:02	B*08:01<br>B*53:01	C*06:02<br>C*07:01
6	30	Male	A*02:01<br>A*25:01	B*35:01<br>B*44:03	C*04:01<br>C*16:01
7	41	Male	A*02:01<br>A*23:01	B*15:01<br>B*44:03	C*04:01<br>C*04:01
8	28	Male	A*02:01<br>A*29:02	B*40:01<br>B*44:03	C*03:04<br>C*16:01
9	41	Male	A*02:01<br>A*29:02	B*08:01<br>B*40:01	C*03:04<br>C*07:01
10	30	Male	A*03:01<br>A*24:02	B*15:10<br>B*35:01	C*03:04<br>C*04:01
11	34	Male	A*02:01<br>A*24:02	B*35:01<br>B*40:02	C*03:04<br>C*04:01
12	37	Male	not tested	not tested	not tested
13	51	Female	A*33:01<br>A*68:01	B*14:02<br>B*48:01	C*08:01<br>C*08:02
14	25	Female	A*01:01<br>A*30:02	B*08:01<br>B*27:05	C*02:02<br>C*07:01
15	42	Male	A*11:01<br>A*30:02	B*35:01<br>B*44:02	C*04:01<br>C*05:01
16	40	Male	A*01:01<br>A*29:02	B*08:01<br>B*49:01	C*07:01<br>C*07:01
17	26	Male	A*02:01<br>A*24:07	B*15:13<br>B*35:05	C*04:01<br>C*08:01
18	41	Male	A*11:01<br>A*29:02	B*51:01<br>B*51:01	C*03:03<br>C*16:01
19	26	Female	not tested	not tested	not tested
20	69	Male	A*01:01<br>A*01:01	B*08:01<br>B*13:02	C*07:01<br>C*07:01
21	39	Female	A*03:01<br>A*31:01	B*07:02<br>B*27:05	C*02:02<br>C*07:02
22	34	Male	A*02:06<br>A*03:01	B*35:01<br>B*45:01	C*04:01<br>C*06:02
23	33	Female	A*01:01<br>A*29:02	B*13:02<br>B*51:01	C*02:02<br>C*06:02
24	53	Male	A*29:02<br>A*30:01	B*42:01<br>B*49:01	C*07:01<br>C*17:01
25	39	Male	A*02:01<br>A*03:01	B*57:04<br>B*58:02	C*18:02
26	19	Female	A*01:01<br>A*03:01	B*08:01<br>B*35:01	C*04:01<br>C*07:01
27	36	Male	not tested	not tested	not tested
28	22	Female	A*02:01<br>A*03:01	B*35:01<br>B*45:01	C*06:02<br>C*16:01
29	24	Female	A*03:01<br>A*66:01	B*35:01<br>B*39:01	C*04:01<br>C*04:04
30	48	Male	A*24:02<br>A*31:01	B*15:01<br>B*18:01	C*03:03<br>C*07:01
31	29	Female	A*11:01<br>A*68:01	B*27:05<br>B*39:02	C*01:02<br>C*07:02
32	28	Male	A*01:01<br>A*02:01	B*57:01<br>B*57:01	C*06:02<br>C*06:02
33	40	Male	not tested	not tested	not tested
34	30	Male	A*02:01<br>A*24:02	B*39:05<br>B*51:01	C*02:02<br>C*07:02
35	38	Male	A*02<br>A*30	B*07<br>B*35	not tested
36	48	Male	A*03:01<br>A*68:02	B*07:02<br>B*14:02	C*07:02<br>C*08:02
37	51	Male	A*01:01<br>A*32:01	B*07:02<br>B*14:02	C*07:02<br>C*08:02
38	51	Male	A*02:01<br>A*02:01	B*40:01<br>B*49:01	C*03:04<br>C*07:01
39	40	Male	A*01:01<br>A*29:02	B*44:03<br>B*57:01	C*06:02<br>C*16:01
40	21	Female	A*24:02<br>A*30:01	B*15:03<br>B*40:02	C*02:10<br>C*03:04
